# Editorial: #ArtGenetics - looking at art to trace plant evolution

**DOI:** 10.3389/fpls.2023.1191677

**Published:** 2023-04-19

**Authors:** Ive De Smet, David Vergauwen

**Affiliations:** ^1^ Department of Plant Biotechnology and Bioinformatics, Ghent University, Ghent, Belgium; ^2^ VIB Center for Plant Systems Biology, Ghent, Belgium; ^3^ Cedom-Madoc, Brussels, Belgium

**Keywords:** art, genetics, evolution, interdisciplinary approach, domestication

Our fruits, vegetables, and cereal crops stem from a wild ancestor and have undergone major changes through millennia of domestication and selection. The approach of combining art history and genetics, or #ArtGenetics, to trace plant evolution and domestication is a fascinating and interdisciplinary field of research. By analyzing artistic and historical representations of crops, researchers can gain insights into the origins, diversity, and distribution of these plants. This approach can lead to the development of new hypotheses and inform future studies. The mini review by De Smet and Vergauwen discusses this approach from the art historian’s point of view and highlights the advantages and limitations of using art history in combination with genetics to study plant evolution. They also call for the development of a global art database to facilitate such analyses.

Kazemi-Shahandashti et al. review the place and time of saffron domestication and cultivation, and address its presumed autopolyploid origin involving cytotypes of wild *Crocus cartwrightianus*. Their article exemplifies the #ArtGenetics approach by using ancient arts and recent genetics to trace the evolutionary origin of saffron crocus.

To map the evolution and domestication of plants, artistic and historical images are a valuable source. This can lead to new hypotheses for researchers studying the origins, diversity, and distribution of a crop. Goldman and Janick discuss artistic and historical representations of table beet to explore the evolution of its root morphology. Myers et al. review 16th-century manuscripts and illustrations to address what and when common beans reached Europe. They suggest that both Middle American and Andean types were in the Caribbean at the time of the Columbian exchange and that beans from both centers were informally introduced into Europe early on. These two examples demonstrate how the analysis of artistic and historical representations can be used to explore the evolution and domestication of crops.

Finally, Arias et al. use a combination of developmental studies and transcriptomics to understand the vegetative domestication syndrome of kale. To identify candidate genes that are responsible for the evolution of domestic kale, they searched for transcriptome-wide differences among three vegetative *B. oleracea* morphotypes. This work further highlights the potential of integrating different approaches in plant evolution research.

Overall, the #ArtGenetics approach represents a promising avenue for studying plant evolution and domestication. As more artwork and historical representations become digitized and easily accessible, it is likely that this field will continue to grow and produce new insights.

## Author contributions

All authors listed have made a substantial, direct, and intellectual contribution to the work and approved it for publication.

